# Socioeconomic Inequalities in COVID-19 Vaccine Hesitancy and Uptake in Greece and Cyprus during the Pandemic

**DOI:** 10.3390/vaccines11081301

**Published:** 2023-07-31

**Authors:** Ioanna Irini Pouliasi, Andria Hadjikou, Konstantina Kouvari, Alexandros Heraclides

**Affiliations:** Department of Health Sciences, School of Sciences, European University Cyprus, 6 Diogenis Str., 2404 Engomi, P.O. Box 22006, 1516 Nicosia, Cyprus; ip222393@students.euc.ac.cy (I.I.P.); a.hadjikou@external.euc.ac.cy (A.H.); kk222533@students.euc.ac.cy (K.K.)

**Keywords:** vaccine hesitancy, vaccination coverage, COVID-19, socioeconomic factors, inequality, population-based survey

## Abstract

Despite the rigorous investigation of the phenomenon of vaccine hesitancy and refusal during the COVID-19 pandemic, the socioeconomic determinants of this phenomenon remain poorly investigated on a global scale. Following proportional quota sampling, we conducted a population-based cross-sectional study. We recruited participants on-site and online from different settings, regions, and socioeconomic strata in two Eastern Mediterranean populations, Greece and Cyprus. Our approach provided a nationwide sample (n = 576) approaching the adult population structure of the two countries, with a slight underrepresentation of men and older people. Our results indicate clear socioeconomic differences in vaccine hesitancy and vaccination coverage, consistent with wider social inequalities in health. In particular, we reveal a clear socioeconomic gradient characterized by lower vaccine hesitancy and higher vaccination coverage, with increasing educational attainment and income. Additionally, participants residing in semi-urban areas show higher vaccine hesitancy and have lower vaccination coverage than those residing in urban and rural areas. Our results could inform Public Health approaches aiming to tackle the alarming phenomenon of vaccine hesitancy by enabling the targeting of population groups who are particularly vaccine-hesitant, rendering such approaches more targeted and effective while at the same time reducing inequalities in the control and prevention of infectious diseases.

## 1. Introduction

Since the emergence of the COVID-19 pandemic in December 2019, about 7 million deaths have been recorded worldwide [1]. Although vaccination plays a crucial role in global Public Health and is the main driver for the eradication of several, but not all, infectious diseases, a substantial proportion of people worldwide are not convinced regarding the necessity and safety of vaccines and remain hesitant [2,3]. Vaccine hesitancy, the phenomenon of refusing or delaying vaccination when necessary and despite the availability of safe and regulated vaccines, is a major obstacle to preventing the spread of infectious diseases [4]. According to the WHO, about 5 billion individuals received the primary COVID-19 vaccination regime globally [5]. However, this has not been adequate, and the phenomenon of vaccine hesitancy has resulted in thousands of preventable deaths during the three-year duration of the pandemic [6,7]. Concentrating in the European Union region, about 45% of adults did not receive the full COVID-19 vaccination regime, while only 15% received the complete regime [8]. In the two countries where the current study focuses, Greece and Cyprus, about 35% of the population remained unvaccinated during the pandemic, skipping the required booster doses [8]. 

Vaccine hesitancy has previously been linked to concerns regarding safety, a presumable high risk of adverse effects, and concerns about low vaccine efficacy [9]. Additionally, mistrust in official healthcare information [10] and general political mistrust [11] have been linked to higher vaccine hesitancy. Additionally, extensive misinformation and disinformation, particularly through certain social media platforms, termed ‘infodemic’ in a pandemic context by the WHO [12], appear to play a crucial role in fueling vaccine hesitancy. Furthermore, vaccine hesitancy has also been detected among healthcare workers [13], who are themselves responsible for promoting and administering vaccines [14], further complicating the issue of reduced vaccine uptake. 

COVID-19 vaccine hesitancy has previously been linked to various demographic and socioeconomic factors. Specifically, socioeconomic factors, such as lower income and residency in deprived or rural areas, have been linked to lower vaccination uptake in a recent systematic review [15]. Similar findings have been identified sporadically in individual studies, with people of lower educational attainment [16], lower employment status [17], and residing in rural areas [18] being more vaccine-hesitant. 

This association between socioeconomic factors and vaccine hesitancy has not been consistent in all populations investigated [19]. Differences in demographic and epidemiological characteristics, economic and infrastructure circumstances, health system structure, and legal and political aspects in different countries were found to influence the process of universal and fair vaccination. This gives rise to differential influences of socioeconomic circumstances between countries [15]. The role of socioeconomic factors in vaccine hesitancy and refusal also appears to be influenced by cultural circumstances [20,21,22] unique to each population. Focusing on the Mediterranean region, lower educational attainment, a worsening economic situation, and rural living were associated with higher vaccine hesitancy and refusal among Italian adults [23,24,25]. Although the phenomenon of vaccine hesitancy has been previously investigated in the Eastern Mediterranean region [26,27,28,29], socioeconomic determinants are usually overlooked. A comprehensive literature review on vaccine hesitancy concluded that the phenomenon was more pronounced in Eastern Europe and the Middle East [30]. In contrast, evidence of socioeconomic inequalities in vaccine acceptance in these regions is scarce [15]. 

Given the above, a research gap exists given the scarcity of evidence on socioeconomic determinants of vaccine hesitancy and uptake in different countries and regions around the world, including the Eastern Mediterranean region. This study aims to contribute to this direction by reporting findings from a population-based survey in two countries from this region, Greece and Cyprus, revealing clear socioeconomic inequalities in COVID-19 vaccine hesitancy and coverage. 

## 2. Materials and Methods

### 2.1. Study Setting and Participant Recruitment 

We conducted a cross-sectional epidemiological observational study in two high-income Eastern Mediterranean European countries, Greece and Cyprus, from early March 2022 to mid-April 2023. The study population consisted of 576 participants over the age of 18, who lived in Greece or Cyprus in the last 3 months, could read in the Greek language, and gave their signed informed consent to participate in the study. Due to the aggravated epidemiological situation due to the COVID-19 pandemic restrictive measures in the countries where the survey was conducted, the sample was selected by sampling predetermined strata (proportional quota sampling). Specifically, a sample of an equivalent percentage to the corresponding proportion of each stratum according to the last census of the population of Greece and Cyprus [31,32] was taken for gender, age, and geographical region (three strata). The sampling units for each stratum were selected non-randomly, in person, or online. The sampling approach followed was identical in the two countries, and no country-specific differences or obstacles were noted, presumably due to the very similar societal structures and cultures of the two countries. 

Probabilistic (random) sampling is the ideal approach in any epidemiological survey. However, it has not been possible to apply such an approach due to the adverse pandemic-related societal circumstances in the two included countries during the data collection stage, amid stringent containment measures and total universal lockdown for all citizens. Following guidelines from the literature regarding achieving roughly representative samples in epidemiological studies, we chose proportional quota sampling as an acceptable alternative to random sampling approaches, anticipated to reduce selection bias [33,34,35]. Such obstacles to sampling have been faced by numerous population-based studies conducted during the pandemic, with some relying on non-ideal approaches such as convenience sampling [36,37,38,39,40]. On the contrary, our sampling approach of choice, proportional quota sampling, has been proposed as an alternative method for obtaining representative population samples during the pandemic, using both in-person and online participant recruitment cost-effectively without compromising study validity if properly conducted [41,42,43].

The target population proportions for the main strata were the following based on the latest census of each country (total population: Greece 10,816,286, The Republic of Cyprus 918,100) [31,32]: Gender (both Greece and Cyprus: 51% women); age-group (Greece: 34% 20–39 years, 34% 40–59 years, 31% ≥60 years; Cyprus: 42% 20–39 years, 34% 40–59 years, 24% ≥60 years); geographical region (Greece: 36% Attica, 28% North Greece, 25% Central Greece, 11% Aegean Islands, and Crete; Cyprus: 38% Nicosia, 28% Limassol, 17% Larnaca, 11% Paphos, 6% Ammochostos).

Participants were recruited from all major regions of Greece (Attica, North Greece, Central Greece, the Aegean Islands, and Crete) and Cyprus (Nicosia, Limassol, Larnaca, Paphos, and Ammochostos), based on the European NUTS classification [44], giving rise to a nationwide sample in both countries. A systematic approach was followed to ensure that selection bias was minimized as much as possible, recruiting participants from different locations and among different socioeconomic strata in order to achieve representativeness. For example, urban, semi-urban, and rural regions were chosen, while in Greece, our sampling was conducted on both the mainland and the islands. Similar stratified and targeted sampling approaches have been followed in similar nationwide studies during the pandemic [45].

In order to determine the minimum sample size required to evaluate our main associations of interest, namely those between socioeconomic factors and vaccine hesitancy, we performed power analysis using the package *pwr* from the R software environment. For this purpose, we assumed a four-category independent variable (e.g., educational attainment or income), a small-to-moderate association (Cohen’s w of 0.20) with vaccine hesitancy as a numeric outcome variable, a significance level of 0.05, and a power of 0.90. This analysis estimated a minimum of 360 participants for determining mean differences of small-to-moderate size in COVID-19 vaccine hesitancy between categories of the major socioeconomic factors analyzed (education and income). Our sample size of 576 participants exceeds the minimum requirement, indicating that our cross-sectional study was adequately powered to detect the main associations of interest. The final sample size of 576 participants was reached after the termination of data collection in April 2023, as a result of the containment of the spread of COVID-19, to a large extent, in the two countries of interest. 

### 2.2. Questionnaire and Data Collection

The questionnaire used was developed for the purposes of the present study. It included questions on sociodemographic factors, COVID-19 vaccine uptake, and other questions relevant to perceptions and intentions toward vaccination (further details in subsequent sub-sections). In order to pilot test the questionnaire, we recruited an initial sample of 69 participants, who provided feedback regarding intelligibility, including comprehension and cultural appropriateness of questions, as well as length (time required to complete) and overall layout of the questionnaire. The pilot testing did not reveal any issues I any of the questions included. 

The first questionnaire section assessed data on demographic and socioeconomic characteristics such as gender, age, country of origin and residence, area of residence (e.g., specific city/town/village), marital status, educational attainment, and monthly income. 

The second section assessed COVID-19 vaccination uptake (number of received doses) and vaccine hesitancy. A COVID-19-specific vaccine hesitancy scale was derived as a composite score based on the following: (i) number of doses received; (ii) short-term intention to get vaccinated; (iii) long-term intention to get vaccinated; (iv) voluntary or forced vaccination; and (v) feeling of satisfaction after getting vaccinated. Further details on the assessment of COVID-19 vaccine hesitancy can be found in the subsequent sub-section. 

In order to evaluate the face validity of key sections of the questionnaire, namely socioeconomic characteristics and vaccination-related information, we also conducted a series of focus groups (6 in total, comprising 10–12 participants each) among this initial sample. Further details about face validity can be found in subsequent sub-sections. Following the pilot testing of the questionnaire, in which no issues were identified, and after confirmation of the face validity of key assessments, the questionnaire was administered in both hard copy and electronic form (created via Microsoft Forms). The questionnaire took approximately 15 min to complete. 

Prospective participants were identified online or in person through targeted sampling. This process continued until the percentages of gender, age, and geographical area within the sample approached the corresponding percentages in each general population (Cyprus and Greece). Both the online and in-person participant recruitment was conducted with the help of postgraduate research assistants who visited places where there was a large transit of people (i.e., large shopping centers, hospitals, supermarkets, and central city/town/village squares). Potential participants were approached by research assistants and were either provided with the print version of the questionnaire or, if the participant requested it, the link to the online version. The print version was usually filled out on the spot, while the online version was filled out by participants on their own time. Responses in the electronic version of the questionnaire were automatically collected in the form of a dataset via the Microsoft Forms platform. Participant recruitment took place from both urban, semi-urban, and rural areas, as well as from more affluent and less affluent areas within major cities. 

In order to estimate the response rate, research assistants were requested to record the number of completed questionnaires and the total number of participants approached. Since data collection was conducted amid the pandemic’s strict restrictive measures and in order to reduce the high burden of data collection, it was conducted only for the first 100 participants approached. Among those, 52 agreed to participate and completed the questionnaire, giving a response rate of 52%. Given that non-response did not alter during the duration of the study, we suggest that this estimation accurately reflects the overall response rate throughout data collection.

### 2.3. Assessment of Main Exposures and Outcomes 

The study questionnaire included an assessment of key socioeconomic indicators, which were treated as the main exposures of interest. Questions on these indicators were phrased and recorded based on the national census of each of the two countries of interest (Greece and Cyprus). 

Area of residence was derived from self-reported city/town/village of residence, which were categorized based on local demographic criteria as: (i) urban (urban areas of more than 10,000 residents); (ii) semi-urban (non-urban areas between 2000–10,000 residents); (iii) rural (non-urban areas with less than 2000 residents). 

Educational attainment was assessed as the highest qualification acquired based on pre-defined categories (Elementary school, Primary school, Secondary school, College, University—Undergraduate level; University—Masters level; University—Doctoral level). For analysis purposes, these categories were re-grouped into the following four-category variable: (i) Up to High School; (ii) College Degree; (iii) Undergraduate University Degree; and (iv) Postgraduate University Degree. 

Participant income was assessed as the typical monthly income, based on pre-defined categories (<500 €/month, 501–1000 €/month, 1001–1500 €/month, 1501–2000 €/month, 2001–2500 €/month, >2500 €/month). For analysis purposes, these categories were re-grouped into the following four-category variable: (i) <500 €/month; (ii) 500–1500 €/month; (iii) 1501–2500 €/month; (iv) >2500 €/month.

COVID-19 vaccine hesitancy and uptake were the outcome variables of interest. The following approach was used for deriving the COVID-19 vaccine hesitancy scale: participants missing 1 dose (5 hesitancy points), participants missing 2 doses (10 hesitancy points), and participants missing 3 doses (15 hesitancy points). Additionally, 1–5 points were added based on the answers to the following 5-item Likert scale questions: (i) “If you were not vaccinated against COVID-19, do you intend to do so?” (ii) “If you were vaccinated against COVID-19, was this with your initial intention or were you forced to do so?” (iii) “If you were vaccinated against COVID-19, how do you feel about this action?” (iv) “If your country adopts long-term vaccination (e.g., every year), based on guidelines from international organizations (e.g., the World Health Organization), for total long-term control of COVID-19, do you intend to follow them?” The derived COVID-19 vaccine hesitancy scale ranged from a minimum of three hesitancy points for participants receiving all three vaccine doses and answering very positively to all questions pertaining to initial intention, perceived satisfaction, and long-term COVID-19 vaccination. On the other hand, it reached a maximum of 25 hesitancy points in participants receiving no doses who answered that they would definitely not get vaccinated either in the near future or in the long term, in case this was required. This variable was used as the main outcome of interest in our analyses. 

In order to investigate the face validity of our COVID-19 vaccine hesitancy scale, we compared the qualitative data collected from the focus groups to the self-reported information gathered through the questionnaire. In particular, the discussion during the focus groups revealed a clear theme characterized by mistrust in authorities and obvious misinformation, with this group of individuals (n = 13) expressing major concerns regarding a presumable exaggeration of the seriousness of the COVID-19 pandemic, with some even denying the existence of the virus. This group of individuals also expressed extreme levels of vaccine hesitancy, claiming that they would definitely not get vaccinated against the specific virus. We labeled these individuals ‘pandemic and vaccination deniers,’ a term very commonly used by the public in both countries of interest. 

In particular, the face validity of the quantitatively assessed (based on relevant questionnaire responses, as noted above) COVID-19 vaccine hesitancy scale was evaluated by comparing the mean score among those identified as ‘pandemic and vaccination deniers’ based on the focus group interviews (mean score = 19.00) to those not identified as such (mean score = 11.03). This difference in the mean COVID-19 vaccine hesitancy score was highly statistically significant (Mann–Whitney–Wilcoxon Test, *p* = 0.003).

A binary variable, termed ‘incomplete COVID-19 vaccine uptake,’ defined as <3 self-reported COVID-19 vaccine doses throughout the COVID-19 pandemic. This variable was additionally used as a secondary outcome in our analyses.

### 2.4. Ethical Issues

All research participation procedures were approved by the Cyprus National Bioethics Committee (EEBK OP 2022.01.36). Participation in the survey was anonymous, voluntary, and without coercion. Moreover, all participants were aware of the aims and scope of the study, the time of completion of the questionnaire, and how the data would be managed, stored, analyzed, and evaluated. They were also made aware of their right to withdraw their participation from the study at any time. At the same time, the platform through which the version of the questionnaire was administered did not record any identifier of the participant (name, contact details, IP addresses, etc.) except for the date and time of submission. The data collected was anonymous, and to ensure the rights and confidentiality of the participants, the electronic files were kept password-protected, with only the principal investigator having access to the data. 

### 2.5. Data Analysis

Descriptive statistics were calculated for a series of sociodemographic characteristics of interest (categorical variables) by country of residence (binary variable—Greece/Cyprus). The chi-squared test was used to investigate the independence between each set of categorical variables (sociodemographic factor vs. country of residence), providing a *p*-value. 

For inferential statistics, regression models were run with the categorical socioeconomic factors of interest (area of residence, educational attainment, and monthly income) included in separate models as independent variables and COVID-19 vaccine hesitancy (numeric scale) and vaccination uptake (incomplete vs. complete vaccination uptake) as the dependent variables, respectively. When vaccine hesitancy was used as the outcome of interest, multiple linear regression was the analysis of choice. When incomplete COVID-19 vaccination uptake was used as the outcome of interest, multiple logistic regression was the choice analysis. 

The influence of possible confounding factors (age, gender, marital status, country of residence) on the above associations was investigated by including these as covariates in the aforementioned regression models, providing estimates adjusted for these factors. 

In order to investigate whether the association between socioeconomic factors and vaccine hesitancy differed by country or by major demographic factors, we ran our linear regression models described above, stratifying by country of residence (Greece vs. Cyprus), by gender (men vs. women), and by age group (aged <60 years vs. ≥60 years). We also included an interaction term between each socioeconomic factor of interest (area of residence, educational attainment, and monthly income) and the three aforementioned potential effect modifiers (country of residence, gender, and age group). A significant *p*-value corresponding to each interaction term indicates a statistically significant interaction (effect modification). Since these were secondary analyses, relevant results are presented in Supplementary Material (Tables S1–S3). 

To check the statistical significance of the above-described statistical tests, a *p*-value at the 5% significance level and a 95% Confidence Interval (CI) are reported. All statistical analyses and data processing were performed using the statistical software R, version 4.3.0.

## 3. Results

Table 1 below displays the demographic and socioeconomic characteristics of study participants for the whole sample (n = 576) and by country. 

Our study sample approaches the basic demographic structure of each population in terms of age, with a slight underrepresentation of individuals aged over 60 years (sample proportions for Greece: 43% 20–39 years, 42% 40–59 years, 12% ≥60 years; and for Cyprus: 52% 20–39 years, 34% 40–59 years, 13% ≥60 years) and geographical region for Greece (sample proportions: 36% Attica, 20% North Greece, 25% Central Greece, 19% Aegean Islands and Crete). The sample deviates from the demographic structure of the source population in terms of geographical region for Cyprus (sample proportions: 65% Nicosia, 16% Limassol, 6% Larnaca, 10% Paphos, 3% Ammochostos) and gender in both countries (∼65% women in the sample).

Given the over-representation of women (63% in the sample vs. 51% in the two source populations) and the under-representation of older individuals (13% aged ≥60 years in the sample vs. 31% in the Greek general population and 24% in the Cypriot general population, respectively), we cannot infer whether our findings definitely apply to specific sub-groups of older people and men, who might not be adequately represented in our sample. 

The majority of participants were married (about 60% of the whole sample) and resided primarily in urban areas (about 84% of the whole sample). The majority of participants had a university education (64% in the whole sample), with the proportion being higher in Cyprus than in Greece (*p* = 0.045). The biggest between-country difference in socioeconomic factors was observed for income (*p* < 0.001). In Cyprus, a much higher proportion of participants (39.2%) reported a monthly income of over €2500 compared to Greece (9.4%). This discrepancy can be attributed to the different socioeconomic circumstances in the two countries, with Greece having been through a major economic crisis during the past decade. In fact, the GDP per capita (PPP) in the two countries (Cyprus $49,930 vs. Greece $36,834) [46] and the average full-time adjusted salary per employee (Cyprus €22,734 vs. Greece €15,879) [47] justify the income differences between the two countries observed in our sample. 

Among the whole sample, 101 participants received no vaccine doses (18.6% of the sample), 79 participants received 1–2 doses (14.6%), and 362 participants received 3–4 doses (66.8%). Small differences in vaccination coverage between countries did not reach statistical significance. The proportions observed are consistent with the population vaccination coverage reported in the two countries during data collection (June 2022–April 2023, 67% uptake of 3–4 doses) [8]. This indicates that the study sample accurately represents the vaccination uptake in the two populations under investigation. 

Table 2 above presents findings from a linear regression model with socioeconomic factors as exposures and COVID-19 vaccine hesitancy as a numeric scale outcome. Starting with an area of residence, it appears that those residing in rural areas do not substantially differ from those residing in urban areas (the reference category) in terms of vaccine hesitancy. However, those residing in semi-urban areas show much higher hesitancy (mean difference, 95% CI: 4.27, 1.16, 7.38). This association was apparent both in Greece and Cyprus, although in Cyprus, those residing in rural areas also had increased vaccine hesitancy (Table S1). None of the tested interactions by country reached statistical significance (Table S1). When stratified by gender and age group, the increased vaccine hesitancy observed among those residing in semi-urban areas was particularly apparent among men (Table S2) and individuals younger than 60 years (Table S2). 

In terms of educational attainment, there is a clear, statistically significant, inverse association with vaccine hesitancy, indicating increasing hesitancy levels with each step up the educational attainment ladder from a College degree and above. This inverse social gradient was apparent in both Greece and Cyprus (Table S1) and slightly more pronounced among men than women (Table S2). Interestingly, educational attainment showed no association with vaccine hesitancy among older individuals (Table S3). 

Monthly income revealed a similar inverse social gradient, with a statistically significant decreasing trend in vaccine hesitancy with increasing income levels above €1500. Although apparent in both countries, this inverse association was more pronounced in Greece than in Cyprus (Table S1) and among men than among women (Table S2). As for educational attainment, income does not appear to be associated with vaccine hesitancy among older individuals (Table S3). 

Table 3 above presents findings from a logistic regression analysis with socioeconomic factors as exposures and COVID-19 vaccine uptake as a binary outcome. Odds ratios represent the likelihood of incomplete vaccination uptake by categories of each socioeconomic factor. These findings corroborate those from Table 2, indicating a higher likelihood of incomplete vaccination uptake among those from semi-urban areas and a decreasing trend of incomplete uptake with increasing educational attainment and monthly income. Differences in the extremes of educational attainment and income reach statistical significance. 

## 4. Discussion

This is the first study to report clear socioeconomic inequalities in vaccine hesitancy and COVID-19 vaccination status in Greece and Cyprus. We reveal a clear inverse social gradient by educational attainment and income on vaccine hesitancy and vaccination coverage, characterized by decreasing hesitancy and higher coverage with increasing socioeconomic position. We also report a novel finding, revealing that in the two populations under investigation, people residing in semi-urban areas (non-urban settings with a population between 2000 and 10,000 residents) have increased vaccine hesitancy and lower uptake of COVID-19 vaccines. These findings are generally consistent in both countries under investigation, are slightly more pronounced among men than women, and are not apparent among older individuals. 

### 4.1. Comparison to Similar Studies in Other Populations 

Our results are consistent with previous evidence suggesting socioeconomic differences in vaccination uptake and hesitancy during the COVID-19 pandemic [15]. Since such socioeconomic stratification of vaccine hesitancy and refusal is context-specific and differs from region to region [15], we confirm that the type of socioeconomic gradient observed in the two Eastern Mediterranean populations under investigation is characteristic of what was observed in high-income European countries and North America. The aforementioned social stratification is characterized by higher vaccine hesitancy and lower uptake of available vaccines among individuals from lower socioeconomic strata (e.g., those with lower educational attainment and receiving relatively lower income) and those residing in non-urban areas. For example, a nationally linked data study in England revealed that vaccination uptake was lower in more deprived areas and among lower socioeconomic groups [48]. Similarly, inequalities in vaccination uptake and intent were observed among various sociodemographic groups in the Canadian Community Health Survey 2021, particularly concerning low educational attainment and region of residence [49], while in a large-scale US survey, vaccine hesitancy was strongly determined by employment status [17]. 

Interestingly, the majority of evidence on socioeconomic inequalities in COVID-19 vaccine hesitancy and uptake follows the above pattern [15]. However, a study of another Mediterranean population, namely Italy, revealed slightly different patterns. In this case, they observed a U-shaped association between educational attainment and COVID-19 vaccine uptake, with decreasing uptake in the highest educational attainment group [50]. Along the same lines, the specific study identified the lowest vaccine uptake in low-density rural areas rather than intermediate-density areas, as identified in the present study. These regional differences probably stem from different characteristics in the analyzed populations. For example, intermediate-density areas in the aforementioned Italian study include large towns and city suburbs, which are probably sociodemographically different from the semi-urban regions in Greece and Cyprus included in the present study, which have a more rural character. 

### 4.2. Potential Explanations for the Inverse Social Gradient in Vaccine Hesitancy and Uptake 

Inverse social gradients in adverse health-related outcomes (such as vaccine hesitancy and refusal) have been repeatedly documented in the literature with different health outcomes [51] as well as health-related behaviors [52]. Different models have been proposed for explaining these social inequalities, including (i) the materialist model, suggesting that inequalities are partly explained via differences in material conditions, amenities, and a deprived built environment [53]; (ii) the cultural–behavioral model, suggesting that inequalities are partly explained via differences in health-related behavior and attitudes [53]; (iii) the access to healthcare model, suggesting that inequalities are partly explained via differences in access, utilization, and uptake of health services, characterized by the phenomenon of the ‘inverse care law’ [54]; and (iv) the psychosocial model, suggesting that inequalities are partly explained by differences in exposure to psychosocial stressors in the working environment and everyday life, involving aspects such as job strain, effort-reward imbalance, and social isolation [55]. 

Our study only presents initial findings on the phenomenon of socioeconomic inequality in vaccine hesitancy and uptake. It does not include any results on explaining this effect via mediation analysis. However, it could be speculated that all of the above-suggested models could contribute to the observed phenomenon. For example, vaccination coverage has been found to be lower in more deprived areas [56] (materialist model). In contrast, vaccine-hesitant individuals have been found to follow specific health behaviors [57] (cultural–behavioral model). Similarly, compromised access to health care [58] and an adverse psychosocial environment [59] have also been linked to higher vaccine hesitancy and refusal. From the aforementioned models, it could be speculated that the most likely scenario is that individuals from lower socioeconomic strata in the two analyzed populations may have a cultural–behavioral profile associated with lower health literacy [60], rendering them more prone to misinformation and institutional mistrust, which have been linked to vaccine hesitancy in the specific populations [61,62]. Material deprivation and reduced access to health care are anticipated to play a minor role in socioeconomic inequalities in this case since the vaccination programs put in place in both countries have been well-organized, systematic, widespread, and free of charge [27,63].

Interestingly, stratified analysis revealed that socioeconomic inequalities in vaccine hesitancy differ slightly in the two countries under investigation, with educational attainment appearing as a more important indicator in Cyprus and monthly income appearing more important in Greece. This might be expected given the differing distribution of income in the two populations, with a large proportion (~40%) of the Cypriot sample belonging to the high-income category (>€2500/month). Based on this finding, income might be a more important socioeconomic indicator in relatively less affluent populations. Socioeconomic differences in vaccine hesitancy were also more apparent among men than women and among younger (<60 years of age) individuals. The latter finding could be explained by the fact that older individuals were under imminent threat from the virus. Therefore, it would be logical and expected that the elderly from all socioeconomic strata would be keen to get vaccinated, hence the lack of any observed association. 

Regarding the area of residence, our results agree with the existing literature that individuals residing in non-urban settings are more prone to vaccine hesitancy [25]. However, we show that this phenomenon is more pronounced in semi-urban than rural areas, particularly in Greece. This could be country-specific and might be explained by the fact that in the two populations under investigation, rural areas are usually populated by older people who are generally less vaccine-hesitant [64]. It could be speculated that a combination of relatively younger ages and low educational attainment, which characterize semi-urban regions in the analyzed populations, might be driving the higher vaccine hesitancy observed in residents of such regions compared to residents of both urban and rural regions.

As in the case of educational attainment and income, the increased vaccine hesitancy in semi-urban areas is more pronounced among men and younger individuals. 

### 4.3. Strengths, Limitations, and Novely of the Present Study 

The strengths of our study include the recruitment of nationwide samples from two populations. In these populations, the phenomenon of vaccine hesitancy and refusal has only been investigated either regionally or based on convenience samples, making recruiting participants accessible to researchers conducting these studies [26,61,62,65]. These previous studies are usually not in the appropriate depth [66] or do not systematically investigate social inequality [28,67]. In contrast, our approach involves the recruitment of participants from different settings, regions, and socioeconomic strata. This provides nationwide samples from both countries of interest, allowing a comprehensive and systematic evaluation of socioeconomic determinants. To our knowledge, this is the first study systematically investigating and revealing clear evidence of socioeconomic inequalities in vaccine hesitancy and uptake in the two analyzed populations (Greece and Cyprus) and the wider Eastern Mediterranean region.

Our study also has some limitations, which should be clarified. Firstly, although we followed a stringent sampling methodology applying proportional quota sampling based on recommended procedures [33,34,35], an approach that actually advocated for the recruitment of roughly representative samples during the pandemic [41], we did not manage to reach all required population strata. Hence, men and older individuals are slightly under-represented in our sample, compromising the generalizability of our findings to some extent. However, we note that the phenomenon of over-representation of women and under-representation of the elderly in population-based surveys is common in the literature. Regarding the former, this has been suggested to stem from gender-specific sociocultural dynamics, such as general altruistic considerations [68], making women more willing to participate in research studies than men [45]. Regarding the under-representation of the elderly, this has also been observed consistently and probably stems from several factors. Older people tend to experience a higher frequency of adverse health conditions, disability, and cognitive decline, which might limit their survey participation. Additionally, a significant proportion of this population group is institutionalized and not accessible for recruitment in population-based surveys [69]. Given this, any stratified results by age group (younger vs. older individuals) presented in the Supplementary Material should be interpreted with caution. due to the relatively low sample size of individuals ≥ 60 years of age (n = 72). 

An additional limitation of our sampling approach is that we were not able to ascertain the response rate throughout the study due to stringent containment measures involving universal lockdowns in both countries under investigation. Therefore, our study’s response rate is estimated based on an initial sample of 100 participants. 

We argue that, given the adverse conditions and the restrictive measures during the COVID-19 pandemic involving strict universal lockdowns in both countries, non-ideal sampling should be expected to some degree. It should be noted that even in long-term national health surveys such as the Health Survey for England, sampling and data collection were adapted during the COVID-19 pandemic to account for imposed restrictions and the sensitivities of potential participants [70]. 

### 4.4. Public Health Implications 

Our findings revealing lower vaccination uptake among population groups with specific socioeconomic characteristics (lower education attainment, lower income, and residing in semi-urban settings) could inform local Public Health programs aiming to achieve the required vaccination coverage in epidemic contexts or regarding routine vaccinations. This could be achieved by tackling more effectively the alarming phenomenon of vaccine hesitancy, identified as a major threat to global health [4], by enabling the targeting of population groups who are particularly vaccine-hesitant, rendering such approaches more tailored and effective. Based on the current findings, such approaches might be particularly effective among men and even more so among younger people than older people. 

Based on the literature, low educational attainment is an almost universal determinant of higher vaccine hesitancy [15]. Therefore, increasing educational attainment and focusing health education resources on less educated groups will probably have a positive effect on vaccination uptake among specific social strata and the population as a whole. Such approaches would simultaneously reduce social inequalities in health, a major global priority according to the WHO [71]. 

Furthermore, although not directly deriving from the current findings, it should be noted that vaccinators themselves (e.g., healthcare workers responsible for administering vaccines) should be educated further in an attempt to reduce the phenomenon of vaccine hesitancy observed among this important population group [13,14]. Not addressing this crucial matter will pose major obstacles for any vaccination campaign among the general public, possibly fueling further health inequalities. 

Finally, it should also be noted that the current findings present a specific snapshot during the COVID-19 pandemic and might not reflect overall attitudes towards vaccinations among the study groups of individuals in a non-pandemic context. In fact, views and attitudes on vaccination in particular population groups studied may change in the post-pandemic era. Given this, there is a need for further studies in order to consistently inform Public Health policy relevant to tackling vaccine hesitancy and improving vaccination uptake. 

In conclusion, for the first time, we report clear socioeconomic inequalities in vaccine hesitancy and refusal in two Eastern Mediterranean populations. We suggest that Public Health approaches aiming to tackle vaccine hesitancy at the population level should not ignore the socioeconomic patterning of the phenomenon, making any attempts more effective and socially fair, helping manage epidemics more efficiently, and reducing health inequalities. 

## Figures and Tables

**Table 1:** Demographic and socioeconomic characteristics of study participants by country of residence.

	Whole Sample (n = 576)	Greece (n = 378)	Cyprus (n = 198)	*p*-Value
**Gender**				
Men	36.5% (210)	36.2% (137)	36.9% (73)	
Women	63.5% (366)	63.8% (241)	63.1% (125)	0.882
**Age group**				
18–30 years	23.6% (136)	21.4% (81)	27.8% (55)	
31–50 years	55.0% (317)	56.3% (213)	52.5% (104)	
51–70 years	17.1% (98)	19.3% (73)	12.6% (25)	
>70 years	4.3% (25)	2.9% (11)	7.1% (14%)	0.110
**Marital status**				
Single	26.9% (155)	28.4% (84)	35.9% (71)	
Married/Cohabiting	66.2% (299)	64.2% (190)	55.1% (109)	
Separated/Divorced/Widowed	6.9% (40)	7.4% (22)	9.1% (18)	0.120
**Area of residence**				
Urban (≥10,000 residents)	84.3% (484)	83.0% (312)	86.9% (172)	
Semi-urban (2000–9999 residents)	5.1% (29)	4.0% (15)	7.1% (14)	
Rural (<2000 residents)	10.6% (61)	13.0% (49)	6.1% (12)	0.014
**Educational attainment**				
High School Diploma	23.8% (137)	25.5% (96)	20.7% (41)	
College Degree	12.2% (70)	14.3% (54)	8.1% (16)	
Undergraduate University Degree	34.9% (201)	33.4% (126)	37.9% (75)	
Postgraduate University Degree	29.1% (167)	26.8% (101)	33.3% (66)	0.045
**Income**				
<€500	14.9% (71)	14.3% (41)	15.9% (30)	
€500–1500	23.3% (111)	30.7% (88)	12.2% (23)	
€1501–2500	40.5% (193)	45.6% (131)	32.8% (62)	
>€2500	21.2% (101)	9.4% (27)	39.2% (74)	<0.001
**Number of vaccine doses**				
0	18.6% (101)	16.4% (62)	86.8% (172)	
1–2	14.6% (79)	4.0% (15)	7.1% (14)	
3–4	66.8% (362)	62.6% (237)	6.1% (12)	0.421

**Table 2:** Mean difference in COVID-19 vaccine hesitancy scale by socioeconomic characteristics of study participants.

	Mean Difference (95% CI) *	*p*-Value
**Area of residence**		
Urban (≥10,000 residents)	reference	
Semi-urban (2000–9999 residents)	4.27 (1.16, 7.38)	0.007
Rural (<2000 residents)	−1.12 (−3.25, 1.00)	0.298
**Educational attainment**		
Up to High School	reference	
College Degree	0.96 (−1.56, 3.48)	0.456
Undergraduate University Degree	−1.87 (−3.65, −0.09)	0.040
Postgraduate University Degree	−4.34 (−6.15, −2.52)	<0.001
**Monthly income**		
<€500	reference	
€500–1500	0.87 (−1.42, 3.17)	0.455
€1501–2500	−1.81 (−3.91, 0.29)	0.090
>€2500	−4.05 (−6.55, −1.54)	0.002

* Estimates derived from a multiple linear regression model, including COVID-19 vaccine hesitancy as the main dependent variable and socioeconomic factors, in turn, as categorical independent variables, adjusting for age, gender, marital status, and country of residence.

**Table 3:** Odds ratios for incomplete COVID-19 vaccination status by socioeconomic characteristics of study participants.

	Odds Ratios (95% CI) *	*p*-Value
**Area of residence**		
Urban (≥10,000 residents)	Reference	
Semi-urban (2000–9999 residents)	2.61 (1.13, 6.28)	0.027
Rural (<2000 residents)	0.72 (0.38, 1.32)	0.297
**Educational attainment**		
Up to High School	Reference	
College Degree	0.73 (0.36, 1.47)	0.386
Undergraduate University Degree	0.54 (0.32, 0.89)	0.017
Postgraduate University Degree	0.34 (0.20, 0.57)	<0.001
**Income**		
<€500	Reference	
€500–1500	1.16 (0.62, 2.18)	0.633
€1501–2500	0.62 (0.35, 1.11)	0.104
>€2500	0.31 (0.15, 0.65)	0.002

* Estimates derived from a multiple logistic regression model, including COVID-19 vaccine uptake (incomplete/complete) as the main dependent variable and socioeconomic factors, in turn, as categorical independent variables, adjusting for age, gender, marital status, and country of residence.

## Data Availability

The data presented in this study are available on request from the corresponding author. The data are not publicly available due to restrictions related to data confidentiality and the prohibition of public sharing, as imposed by the local bioethics committee, which granted approval to the current study.

## Supplementary Materials

The following supporting information can be downloaded at: https://www.mdpi.com/article/10.3390/vaccines11081301/s1, Table S1: Mean difference in COVID-19 vaccine hesitancy scale by socioeconomic characteristics of study participants, stratified by country of residence; Table S2: Mean difference in COVID-19 vaccine hesitancy scale by socioeconomic characteristics of study participants, stratified by gender; Table S3: Mean difference in COVID-19 vaccine hesitancy scale by socioeconomic characteristics of study participants, stratified by age-group.
