# Supplementary material for: Socioeconomic Inequalities in COVID-19 Vaccine Hesitancy and Uptake in Greece and Cyprus during the Pandemic
Source: Vaccines (Basel). 2023 Jul 31;11(8):1301. doi: 10.3390/vaccines11081301 (PMC10459981; doi:10.3390/vaccines11081301)
Supplement: Supplementary file 1 [file vaccines-11-01301-s001.zip › vaccines-2499181-supplementary.pdf]

Table S1. Mean difference in COVID-19 vaccine hesitancy scale by socioeconomic characteristics of study participants, stratified by country of residence.

	Mean difference (95% CI)*			
**Area of residence**	***Whole sample***	***Greece***	***Cyprus***	*p-value for interaction*
Urban (≥10000 residents)	reference	reference	reference	
Semi-urban (2000-9999 residents)	4.27 (1.16, 7.38)	3.56 (-1.05, 8.18)	4.54 (0.37, 8.71)	*0.66*
Rural (<2000 residents)	-1.12 (-3.25, 1.00)	-2.14 (-4.54, 0.26)	3.00 (-1.50, 7.51)	*0.10*
**Educational attainment**				
Up to High School	reference	reference	reference	
College Degree	0.96 (-1.56, 3.48)	0.51 (-2.66, 3.67)	3.00 (-1.21, 7.21)	*0.18*
Undergraduate University Degree	-1.87 (-3.65, -0.09)	-1.44 (-3.78, 0.91)	-1.93 (-4.76, 0.90)	*0.76*
Postgraduate University Degree	-4.34 (-6.15, -2.52)	-3.35 (-5.71, -0.98)	-5.61 (-8.51, -2.71)	*0.47*
**Monthly income**				
<€500	reference	reference	reference	
€500-1500	0.87 (-1.42, 3.17)	0.21 (-2.56, 3.01)	1.97 (-2.22, 6.17)	*0.50*
€1501-2500	-1.81 (-3.91, 0.29)	-3.28 (-5.93, -0.63)	-0.35 (-3.81, 3.10)	*0.17*
>€2500	-4.05 (-6.55, -1.54)	-6.06 (-9.82, -2.30)	-2.18 (-5.96, 1.60)	*0.26*

* Estimates derived from a multiple linear regression model, including COVID-19 vaccine hesitancy as the main dependent variable and socioeconomic factors, in turn, as categorical independent variables, adjusting for age, gender, and marital status, and including an interaction term between socioeconomic factors and country of residence.

Table S2. Mean difference in COVID-19 vaccine hesitancy scale by socioeconomic characteristics of study participants, stratified by gender.

	Mean difference (95% CI)*			
**Area of residence**	***Whole sample***	***Men***	***Women***	*p-value for interaction*
Urban (≥10000 residents)	reference	reference	reference	
Semi-urban (2000-9999 residents)	4.27 (1.16, 7.38)	7.42 (2.08, 12.75)	2.24 (-1.58, 6.06)	*0.18*
Rural (<2000 residents)	-1.12 (-3.25, 1.00)	-0.08 (-3.46, 3.29)	-1.97 (-4.72, 0.78)	*0.25*
**Educational attainment**				
Up to High School	reference	reference	reference	
College Degree	0.96 (-1.56, 3.48)	-0.30 (-4.78, 4.18)	1.69 (-1.39, 4.78)	*0.35*
Undergraduate University Degree	-1.87 (-3.65, -0.09)	-2.09 (-5.06, 0.88)	-1.51 (-3.76, 0.74)	*0.45*
Postgraduate University Degree	-4.34 (-6.15, -2.52)	-5.63 (-8.62, -0.64)	-3.40 (-5.71, -1.09)	*0.11*
**Monthly income**				
<€500	reference	reference	reference	
€500-1500	0.87 (-1.42, 3.17)	1.41 (-3.26, 6.13)	0.15 (-2.48, 2.78)	*0.54*
€1501-2500	-1.81 (-3.91, 0.29)	-1.94 (-6.22, 2.34)	-2.37 (-4.80, 0.06)	*0.61*
>€2500	-4.05 (-6.55, -1.54)	-5.29 (-9.83, -0.76)	-2.42 (-5.55, 0.70)	*0.31*

* Estimates derived from a multiple linear regression model, including COVID-19 vaccine hesitancy as the main dependent variable and socioeconomic factors, in turn, as categorical independent variables, adjusting for age, marital status, and country of residence, and including an interaction term between socioeconomic factors and gender.

Table S3. Mean difference in COVID-19 vaccine hesitancy scale by socioeconomic characteristics of study participants, stratified by age-group.

	**Mean difference (95% CI)***			
**Area of residence**	***Whole sample***	***Younger***	***Older***	*p-value for interaction*
Urban (≥10000 residents)	reference	reference	reference	
Semi-urban (2000-9999 residents)	4.27 (1.16, 7.38)	4.49 (1.08, 7.89)	1.09 (-6.62, 8.81)	*0.68*
Rural (<2000 residents)	-1.12 (-3.25, 1.00)	-0.94 (-3.37, 1.48)	-2.32 (-6.60, 1.97)	*0.83*
**Educational attainment**				
Up to High School	reference	reference	reference	
College Degree	0.96 (-1.56, 3.48)	-0.60 (-3.36, 2.15)	15.99 (7.31, 24.64)	*0.012*
Undergraduate University Degree	-1.87 (-3.65, -0.09)	-2.61 (-4.68, -0.53)	-0.74 (-4.28, 2.80)	*0.81*
Postgraduate University Degree	-4.34 (-6.15, -2.52)	-5.73 (-7.81, -3.64)	-0.12 (-4.85, 4.26)	*0.14*
**Monthly income**				
<€500	reference	reference	reference	
€500-1500	0.87 (-1.42, 3.17)	-0.01 (-2.56, 2.55)	1.52 (-3.89, 6.93)	*0.52*
€1501-2500	-1.81 (-3.91, 0.29)	-3.01 (-5.35, -0.65)	-1.00 (-6.39, 4.39)	*0.15*
>€2500	-4.05 (-6.55, -1.54)	-5.06 (-7.84, -2.28)	-1.10 (-6.92, 4.72)	*0.30*

* Estimates derived from a multiple linear regression model, including COVID-19 vaccine hesitancy as the main dependent variable and socioeconomic factors, in turn, as categorical independent variables, adjusting for gender, marital status, and country of residence, and including an interaction term between socioeconomic factors and binary age (<60 years vs. ≥60 years).
